# Fifty years of progress for beef cattle production in the United States

**DOI:** 10.1093/jas/skag169

**Published:** 2026-06-03

**Authors:** C Alan Rotz, Jose P Castano-Sanchez, Daniel M Kniffen, Matt Buyers, Samantha J Werth

**Affiliations:** USDA-ARS, Pasture Systems & Watershed Management Research Unit, University Park, PA 16802, United States; USDA-ARS, Food Systems Research Unit, Burlington, VT 05405, United States; Animal Science Dept., The Pennsylvania State University, University Park, PA 16802, United States; Agri Beef, Boise, ID 83702, United States; US Roundtable for Sustainable Beef, National Cattlemen’s Beef Association, Centennial, CO 80112, United States

**Keywords:** beef cattle, Integrated Farm System Model, life cycle assessment, carbon, nitrogen

## Abstract

Many changes in feed production and the management and genetics of cattle have occurred over the past 50 years which affect the efficiency, productivity and environmental impacts of beef cattle production. Trends in feed and animal productivity were determined through regression of historical annual data with time. The Integrated Farm System Model, following life cycle assessment procedures, was used to assess the impact of these changes by modeling representative cattle operations throughout the US in 1972 and 2022. Improvements in yields of feed crops, animal management and genetics, machinery efficiency, and the production of resources such as fuel, electricity, and fertilizer have led to many reductions in the life cycle environmental impacts of beef cattle production. Fewer but larger cattle harvested in 2022 produced 23% more beef carcass weight than in 1972. Nearly the same total feed was consumed to produce beef in 2022 as used in 1972, but about 10% less forage and 37% more grain and byproduct feeds were fed. Increases in crop yields, particularly corn yields, led to a 14% reduction in cropland and 11% reduction in total land use over the 50-yr period. Other predicted improvements included a 27% reduction in blue water use, 26% reduction in fossil energy use, and 8% reduction in methane emissions. There was little change in total reactive N losses related to beef cattle production with a 33% increase in ammonia emissions offset by decreases in N denitrification, leaching and runoff losses. Considering the increase in animal weight produced, reductions per unit of carcass weight included 22% less feed, 29% less land, 42% less blue water, 41% less fossil energy, 27% less methane, and 21% less reactive N loss. A sensitivity analysis showed that these results were not influenced much by changes in our assumptions on past animal and pasture management. These improvements reflect the continuing trend of the U.S. cattle industry for increasing productivity and efficiency to produce a sustainable product that meets the growing demand for beef.

## Graphical Abstract

**Graphical Abstract:**
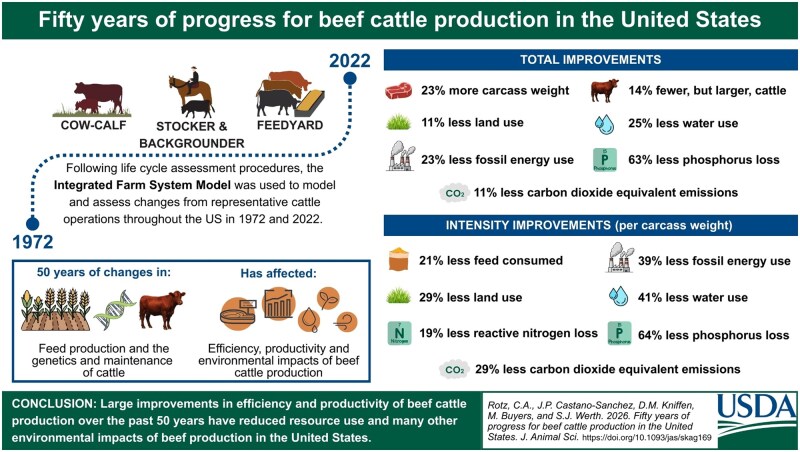

## Teaser

Large improvements in efficiency and productivity were made in beef cattle production over the past 50 years with 23% more beef now produced using fewer cattle. This has reduced the resources used and many other environmental impacts of beef production in the United States.

## Introduction

The past fifty years have seen many changes leading to improved productivity and efficiency of beef production in the US. These include many aspects of feed production as well as the management and genetics of cattle. Major changes have also occurred in the efficiency of producing resources used in cattle production such as fuel, electricity and fertilizer. Machinery size and efficiency have increased along with improvements in fuel efficiency, and the sizes of farms, ranches, and feedlot finishing operations have increased. These changes have led to more efficient production of beef. In a 30-yr analysis comparing 1977 to 2007, Capper (2011) showed that per unit of beef produced in the US, feed consumption decreased by 19%, water use by 12%, and land use by 33%.

Integrating and quantifying the impact of all these changes is challenging. Life cycle assessment (LCA) provides a tool that assists this type of analysis. Life cycle assessment is an accounting procedure that considers all the important processes and their integration over the life of a product or service. This approach has been used to quantify various impacts of individual beef production systems in the US (Stackhouse-Lawson et al. 2012; Lupo et al. 2013; Rotz et al. 2013; Asem-Hiablie et al. 2019; Webb et al. 2020; Castano-Sanchez et al. 2023). Life cycle assessment has also been used to evaluate strategies for mitigating emissions (Pelton et al. 2024) and to determine the uncertainties in quantifying emissions from US beef cattle (Dudley et al. 2014).

Process-level simulation of production systems has been combined with LCA to quantify national impacts of cattle production in the US (Rotz et al. 2019). This was followed by the analysis of Putman et al. (2023) which included processing, retail and the consumer to determine impacts over the full life cycle of beef. By applying this same modeling and LCA procedure to cattle operations in the 1970s, similar information can be determined for cattle production at that time. By comparing past and recent values of performance and related impacts, improvements over this time can be quantified. This type of assessment of US dairy farms has shown substantial improvements in production efficiency with corresponding reductions in environmental impacts of the milk produced (Rotz et al. 2024).

Our objective was to quantify improvements in US beef cattle production from 1972 to 2022 using LCA supported by historical records and process-level simulation of feed and cattle production systems. Assessed changes included cattle performance, feed consumption, fossil energy use, gas emissions, water use, land use, and nutrient losses. Comparisons included both totals over all cattle produced and intensities expressed per unit of carcass weight (CW) produced.

## Materials and methods

To represent and assess current and past beef production in the US, representative operations were modeled and simulated using the Integrated Farm System Model (IFSM; USDA-ARS 2026). Operations included crop farms producing feed, ranches and feedlots producing cattle, and dairy farms producing cull animals used in beef production. Current operations were modeled using the procedure and supporting information documented by Rotz et al. (2019). To model operations in the 1970s, historical data and information were gathered to determine trends for various changes in beef cattle production.

### Integrated farm system model

The IFSM used process-level simulation on a daily time step over many years of weather to predict the performance and environmental impacts of farms, ranches and feedlots. For each operation, the model simulated crop and pasture production, feed use, animal production, and the return of manure nutrients back to the land (Rotz et al. 2022). Daily growth and development of crops, pasture or rangeland were a function of soil water and N availability and weather. Simulated tillage, planting, and harvest operations determined labor, fuel and other resources used, timeliness of operations, crop losses, and nutritive quality of feeds produced. Animal feed intake, growth and nutrient excretion were a function of animal requirements and the nutrient contents of feeds fed.

Nutrients (N, P, K) and C were tracked within the model to predict losses and whole farm balances (Rotz et al. 2022). Paths of N loss included ammonia (NH_3_) volatilization, nitrous oxide (N_2_O), N oxides (NOx) and dinitrogen (N_2_) emissions through nitrification and denitrification processes and engine emissions, and nitrate (NO_3_^−^) losses through leaching and runoff. Carbon emissions included methane (CH_4_) from enteric and manure sources along with biogenic carbon dioxide (CO_2_). Anthropogenic CO_2_ emissions were released through fossil fuel combustion and the decomposition of lime and urea fertilizer. Other simulated losses included erosion of sediment and runoff of sediment-bound and dissolved P across farm boundaries. Reactive volatile organic compound (VOC) emissions were predicted from silage and manure exposed to ambient air. Emission processes were simulated on an hourly or daily time step using dynamic relationships influenced by temperature, wind speed, precipitation, soil conditions, and management practices (Bonifacio et al. 2015; Rotz et al. 2014, 2022). Whole-farm mass balances of N, P, K, and C were the sum of nutrient imports in feed, fertilizer, deposition, and fixation, minus the exports in losses, manure leaving the operation, and feeds and animals sold.

Following the Livestock Environmental Assessment and Performance guidelines (LEAP 2016), a cradle-to-farm gate LCA was performed within IFSM (Rotz et al. 2022). The system boundary was that of the farm, ranch or feedlot. Major components included the soil, feed crops, machinery operations, cattle, animal facilities, and manure handling processes. System inputs or resources used included fuel, electricity, fertilizer, purchased feeds and supplements, machinery, seed, and pesticides. Emission or consumption factors used for these upstream sources were set to reflect those of 1972 or 2022 (Table 1 in Rotz et al. 2024). Resource inputs required and the various emissions were determined through the dynamic simulation. Both the intensity expressed per unit of CW produced and the total of all cattle produced were used as functional units for comparisons between the two points in time. Manure exported from operations was treated as a residual where no impacts associated with manure were allocated to manure leaving the operation. Emissions from manure after removal were also not included in the beef system.

**Table 1:** Cattle weights and average daily gains for typical production life cycles in 1972 and 2022.

	Cow-calf phase				Stocker/background phase			Finish phase			Life cycle	
	Birth weight	Period	ADG	Wean weight	Period	ADG	**Out weight** ^1^	Period	ADG	Out weight	Life	ADG
Year	kg	month	kg/day	kg	month	kg/day	kg	month	kg/day	kg	months	kg/day
**Feedlot backgrounding systems**												
**1972**	33	7	0.81	205	4	0.67	286	5	1.14	460	16	0.88
**2022**	37	6.5	1.18	270	3	0.76	339	6.5	1.45	625	16	1.21
**Short stocker production systems**												
**1972**	33	7	0.81	205	6	0.64	321	4	1.14	460	17	0.83
**2022**	37	6.5	1.18	270	4.5	0.67	362	6	1.44	625	17	1.14
**Long stocker production systems**												
**1972**	33	8	0.65	190	8	0.56	325	5	1.15	500	21	0.73
**2022**	37	7	0.77	200	8	0.80	394	7	1.44	700	22	0.99
**Cow-calf to finish production systems**												
**1972**	33	8	0.71	205	8	0.48	321	4	1.14	460	20	0.70
**2022**	37	7	1.09	270	7	0.85	450	4	1.44	625	18	1.07
**Dairy calf production systems**												
**2022**	42	5	0.91	181	6	1.01	365	6	1.26	595	17	1.07
**2022**	42	4	0.80	139	7	1.02	355	6	1.26	584	17	1.05
**2022**	42	4	0.80	139	2	1.00	200	9	1.25	543	15	1.10

1 Out weight of stocker/background phase is the in weight of the finish phase.

The farmgate LCA determined annual feed consumption, fossil energy use, blue water use, land use, and C and N losses (Rotz et al. 2022). Feed consumption included the total of all pasture, range and concentrate dry matter consumed. Fossil energy included all fuel used in farm, ranch or feedlot operations (machinery, trucks and other vehicles) along with that used to produce electricity and other resources consumed in the operation. Blue water was defined as that from ground and surface sources, which consisted of water used for irrigated crop production and drinking and cooling of cattle. Land use was pasture and rangeland grazed by cattle plus land used to produce additional forage and grains fed. For byproduct feeds, land and other inventories were allocated among crop products based upon economic value.

Annual emissions of CH_4_, N_2_O and anthropogenic CO_2_ were combined as CO_2_ equivalents (CO_2_e) using conversions of 27 g CO_2_e/g CH_4_ and 273 g CO_2_e/g N_2_O (Forster et al. 2021). Emissions included both direct emissions from the production system, as well as indirect N_2_O emissions that occurred elsewhere in the environment resulting from transformation of NH_3_ and NO_3_^−^ lost from the production system (IPCC 2006). Reactive N loss was the total of all forms of reactive N associated with the production of beef cattle lost to the environment.

Other nutrient losses or emissions compared over time included CH_4_, N_2_O, anthropogenic CO_2,_ NH_3,_ NO_3_^−^, and non-methane VOCs. Phosphorus runoff losses across ranch boundaries in both soluble and sediment-bound forms were also considered. These individual emissions were determined as direct losses from ranches and feedlots only, i.e., LCA was not used to include the effects of producing imported resources.

### Historical data

To quantify changes over the 50-yr period, historical annual data were gathered on crop and animal productivity. An important source of data was the national agricultural census and survey data documented by the National Agricultural Statistics Service (USDA NASS 2025). Other annual data were available on cattle weights, gains and feed efficiency (Betts and Harborth 2024; AAA 2025) and fertilizer (USDA-ERS 2025) and pesticide (Fernandez-Cornejo 2014) use on crops. These annual data provided a means of quantifying trends over time.

Trends were determined through regression of annual data with time. In most cases, a linear relationship was used but, in a few cases where the data peaked or leveled at some point through time, a polynomial relationship provided a better fit. The R-squared value for the relationship with time was used as an indicator of the significance of the trend considering the annual variability in the data. An R-squared value greater than 0.7 indicated a strong trend and values less than 0.2 indicated little change or the inability to predict a change over the period. The regression equations developed were used to predict values for each feed and animal characteristic in 1972 and 2022. This method assisted in setting the end points of the 50-yr period by smoothing annual variability. However, in cases such as number of cows harvested where considerable annual variability masked significant changes, we still considered trends and documented data to set values for our LCA that provided balanced production systems.

#### Feed production

Crop production and yield data were available for each year over the 50-yr period for the major feed crops of corn grain, corn silage, sorghum grain, sorghum silage, wheat, alfalfa hay, grass hay and soybeans (USDA NASS 2025; Table S1). The total and irrigated areas harvested and irrigation water applied were also available for each crop to document irrigation use. Annual values for cropland receiving fertilizer and the amount applied were available for corn and wheat crops (USDA-ERS 2025). These data provided trends for changes in N and P fertilizer use. Annual herbicide and insecticide data were available for corn production from Fernandez-Cornejo (2014). These included both types and amounts of chemicals applied along with a total mass of active ingredients applied.

Because water use in feed production was an important consideration (Rotz et al. 2019), special effort was given to quantifying water use in pasture irrigation and purchased feeds. For pasture, census data were available on total pasture and rangeland areas, irrigated pasture area and the water applied from 1969 to 2023 (USDA NASS 2025). These data were used to determine the change in water use over time.

Water used to irrigate purchased feeds was determined by comparing the amount of each feed used to that produced in the state (Rotz et al. 2025, 2026). Amounts of cattle feed consumed in each state were obtained from the Animal Feed Consumption report (DIS 2025). Grain use also included corn and sorghum used in ethanol production (USDA NASS 2025). That used for ethanol was distributed across states based upon distillery capacity (Rotz et al. 2025). In states or regions where more grain was produced than that used for feed and ethanol production, a water footprint for the grain produced was determined as the irrigation water applied divided by the total feed produced in the state. When more grain was used than was produced, a weighted footprint was determined considering the footprint for that produced in the state and that imported from other states (Rotz et al. 2025, 2026). The imported footprint was a national footprint determined as the total water applied nationally divided by the total dry matter of each grain produced in the US.

Similar procedures were used for determining water footprints for purchased forage. For corn silage, the forage was assumed to be grown within the state, so no import occurred. Thus, the water footprint in each state was determined as the total water applied to corn silage land divided by the silage DM produced in the state (USDA NASS 2025). For both alfalfa and grass hays, importation into the region was considered when the amount produced was not sufficient to meet that needed for feed. When imported feed was required to meet the needs within the state, the footprint was determined as the weighted average of that produced in the state and that imported from other states (Rotz et al. 2025).

Land use included the land covered by the operation and that used to produce purchased feeds. Land used to produce feeds imported to the operation was determined as the feed purchased divided by the associated national average crop yield in 1972 or 2022. Stocking rates and associated forage availability on pasture and rangeland in 2022 were calibrated for each state or region considering the survey data of Asem-Hiablie et al. (2015, 2016, 2017, 2018). Pasture and rangeland productivity was assumed to remain similar across the 50-yr period (USDA NASS 2025). Therefore, land used for grazing was set based upon the 500 kg animal units grazed, i.e. the grazing land used per animal unit in 1972 was set similar to that used in 2022. This may have been a conservative assumption, so the sensitivity to stocking rate was evaluated as noted below in the sensitivity analysis.

#### Cattle production

Annual cattle numbers for different types and ages were available from USDA NASS (2025). These included the number of cows maintained and harvested each year for both beef and dairy production. The number of beef-breed heifers maintained was compared to the number of cows to determine an annual replacement rate. Along with cows, the number of bulls, steers, and heifers harvested each year, and their dress weights provided a total CW produced. To separate beef and dairy cattle harvested, the number of beef-breed steers was estimated as half of the weaned calves produced minus those raised as bulls, and the difference between total steers and beef-breed steers provided the number of steers originating from dairy farms (Table S2, supplemental information). Similarly, dairy heifers used for beef were determined as the total heifers harvested minus those from beef breeds and those used as replacements. The recent increase in beef on dairy and the use of sexed semen, may influence the ratio of dairy heifers to steers, but this had little effect on our assessment since the total cattle coming from dairy farms was what was important.

Dress weights were available for all cows, bulls, steers and heifers harvested each year along with the total CW produced (USDA NASS 2025). Based upon the number of dairy cull cows and dairy steers harvested, the total CW produced was divided between dairy and beef-breed cattle. An overall dressing percentage was also provided to indicate the trend for any change over time. To verify the portion of beef originating from dairy farms, the total of all animals and their CW were compared to the total national CW produced to assure that animal numbers and weights balanced with national production.

Annual data on feedlot finishing of cattle were available from 2000 to 2022 (Betts and Harborth 2024). These included cattle in weights, out weights, days on feed, average daily gain, feed intake, gain to feed ratio, dressing percentage and final CW. Highly significant linear trends were found for final CW, days on feed and feed intake. These trends were used to project back to estimates for 1972 where final CW was verified to agree with that obtained from USDA NASS (2025).

### Producer information

Although much of the information needed to model representative operations was available through historical data and information gathered, further information was sought from producers and other experts familiar with cattle production over this time. Three groups of 8 to 10 each were established representing grazing operations in the eastern and western regions and feedlot finishing. The grazing groups included cow-calf, cow-calf to stocker, and cow-calf to finish producers, and the feedlot group focused on backgrounding and finishing of cattle. Each group met through a virtual meeting which was followed by survey questions specific to their operations. Additional correspondence was used as needed.

Information gathered focused on management practices not commonly documented in historical data. For grazing operations, this included changes in pasture and rangeland productivity, stocking rates, supplemental and winter feeding, fertilizer use, pasture renovation, and weaning age. Feedlot information included ages of cattle, use and length of backgrounding, associated cropland for feed production, byproduct feed use, diet forage and protein contents, and manure handling practices. Their expert opinions were considered along with any other data or information they provided when setting parameters for simulated cattle operations. The potential impact of many of these assumptions was determined in the sensitivity analysis.

An important consideration in our assessment was the life cycle of cattle. The production of beef cattle includes cow-calf, stocker or backgrounding and finishing phases (Endres and Schwartzkopf-Genswein 2018), but the time calves spend in the first two phases was not well documented for current or historical production systems. Based upon animal weights, average daily gains, and producer insight, representative production cycles were established for traditional beef cattle and dairy calf production (Table 1). Dairy calves were only considered in 2022 because few cull dairy calves were used for beef production in 1972 when veal production was more prevalent (Table S2; USDA NASS 2025).

### Representative operations

Forty representative operations were simulated in the eastern and western regions of the US to quantify changes in beef cattle production and resulting impacts. Eastern region was defined as all states from Minnesota to Louisiana and east and those west of this line were the western region. Simulated operations in each region included 4 cow-calf, 3 cow-calf to stocker, 2 cow-calf to finish, 2 stocker or background, up to 4 feedlot finishing, 2 Holstein finishing and 2 dairy farms. Parameters were set to describe each operation in 1972 and 2022 using the data gathered. Brief descriptions of the operations are provided in Table S3 of the supplementary information.

To define operations for 2022, a subset of those used by Rotz et al. (2019) were selected to represent each region. Parameter changes were made to update those operations from the 2017 data of the previous study. Some important changes were animal numbers and weights at each stage and total CW produced. Crop yields were also adjusted along with the resulting land area needed to produce the required feed. Environmental intensities for producing purchased resources and feeds were updated to those used by Rotz et al. (2024) to better represent changes over the 50 years.

To represent cattle operations in 1972, the operations used in 2022 were adapted to earlier conditions. Thus, the same locations with the same soil parameters and weather data were used for each. This was supported by USDA NASS (2025) data that indicated that there were no important shifts in the location of cattle production over this period. Given that we used a relatively small number of modeled operations to describe the US beef industry, this also reduced confounding effects that could be introduced by using different weather and soil conditions.

Parameter adaptation included the size of operations, crop and animal characteristics, and other important changes in management. Operation sizes were reduced using a 25% reduction in cattle numbers on ranches and an 80% reduction in the size of feedlot finishing operations (USDA NASS 2025). Weights for various animal types and stages of growth were reduced to that determined for 1972. Average daily gains were also reduced to match the conditions of that time (Ottoson 1975). For finishing operations, feed efficiency was adjusted to ensure the model was properly representing feed intakes and feed to gain ratios (Ottoson 1975; Betts and Harborth 2024). Other adjustments included using more grazing stocker production with less time in feedlot backgrounding. Whereas growth implant, ionophore and beta agonist treatments were used in most current operations (Rotz et al. 2019), they were removed to reflect common practices in the early 1970s. Some adjustment in supplemental feeding was made by reducing the use of byproduct feeds, particularly the use of distiller’s grain, in 1972.

Changes in manure handling practices over the 50 years were minor. For grazing operations, all manure was returned to pastures and rangeland. With less importation of feed in finishing operations in the 1970s, less manure was exported. Runoff catchment basins were also less common. Whereas retention ponds were used on feedlots in 2022 to handle about 10% of the manure nutrients, all manure was managed using stacks in 1972. Active composting of manure was used on a couple feedlots in 2022 but not in 1972.

For farms and ranches in the 1970s, land area was adjusted to provide a similar portion of the feed requirement considering changes in animal numbers and crop and pasture yields. Thus, land area was set in proportion to the difference in cattle numbers and BW and the resulting feed required. The crop area managed by feedlots in the 1970s was assumed similar to that used in 2022. As the size of feedlots increased over the 50-yr period, feed production area managed by the feedlots did not increase in proportion. Thus, less importation of feed and less export of manure nutrients was needed in the 1970s.

Inorganic fertilizer use was adjusted to reflect reported changes over the 50-yr period. For corn, the use of inorganic N fertilizer did not change much over the period despite the large increase in yield, and the use of phosphate fertilizer decreased (USDA-ERS 2025). For grassland and pasture, there was also a decrease in fertilizer use (producer opinion).

To represent machinery in the 1970s, sizes were reduced where appropriate, and the fuel efficiency of diesel engines was reduced by 20% (Rotz et al. 2024). Smaller tractors were also set to use gasoline for fuel with a lower fuel efficiency. Hay production was changed from the current large bale systems to the small bale harvest and storage systems commonly used at that time. This included a change from the use of rotary disk to smaller sicklebar mower-conditioners with a lower harvest capacity. In cases where no-till or reduced tillage was used in current operations, conventional tillage with multiple tillage operations was used in the 1970s. In eastern operations in the 1970s, primary tillage often used a moldboard plow to invert the soil leaving a surface that promoted erosion and runoff.

Because dairy cattle were an important source of beef, dairy farms were also modeled for 1972 and 2022. The four dairy farms used were obtained from the assessment of Rotz et al. (2024). The total farm impacts were allocated between milk production and cull animals based upon the biophysical relationship documented by IDF (2022). The total allocated to beef cattle was split between cull calves and cows according to the annual mass of each leaving the dairy farm and their net energy for growth (IDF 2022). In 1972, cull calves were not used for beef production. In 2022, cull calves were assumed to leave the dairy soon after birth where they were weaned over a few months on a calf rearing facility (Stackhouse-Lawson et al. 2012). Weaned calves then went to a backgrounding and finishing facility to complete their life cycle (Table 1).

To help verify model predictions, simulation results for cattle production in 2022 were compared to those published by Rotz et al. (2019). Life cycle intensities for feed consumption, CO_2_e emissions, fossil energy use, water use and reactive N loss were compared. Fewer operations were used in the current study, so the verification was needed to ensure that they adequately represented the national industry. As stated above, parameters such as animal numbers and weights were updated for 2022, and minor model changes also occurred since the previous study. Considering these differences between studies, direct comparisons could not be made, but the general comparison helped verify that current operation results were similar to those of the previous study.

### Sensitivity analysis

Some of the parameters assumed for 1972 or the difference between 1972 and 2022 were not well defined through documented historical data. A sensitivity analysis was done to measure how inaccuracies in those assumptions would affect the predicted changes over the 50-yr period. Evaluating changes across all operations simulated was not feasible. Instead, single simulations were done representing traditional beef cattle production in 1972 and 2022. For these simulations, cattle numbers were set to 1/10,000 of the beef-breed animals reported for each time, and land and other parameters were scaled accordingly. Management practices were set as most often used in the original simulations for each point in time. Life-cycle data from these single simulations were compared to those found through simulations of the multiple operations to assure that the production of traditional beef cattle for the US was properly represented by the single simulation. Any change related to the increased use of cattle from dairy operations was not included in the sensitivity analysis.

Parameters evaluated encompassed pasture and animal management. Assumptions related to pasture management included differences in stocking rate and pasture yield between 1972 and 2022. For parameters assumed to be the same across the 50-yr period, changes were made in 1972 to create a difference. These included a reduction in pasture quality (10% less CP and 10% greater NDF in 1972), a 20% reduction in sward legume content (5% decreased to 4%), a 15% reduction in pasture irrigation, and a 10% reduction in N fertilizer use.

Several assumptions related to animal management were tested through changes in the 1972 simulation. These included increasing the stocker period by 1 month, increasing the finish period by 1 month, decreasing calf weaning rate 2% (from 84.4% to 82.8 with 2% more cows), increasing protein fed by 10%, and increasing the forage content in finishing diets by 10%. Two additional assumptions were evaluated by changing the difference between 1972 and 2022. Annual cow replacement rate in 1972 was changed from 11 to 12% to provide a 50% reduction in the difference over the period. Differences in purchased feed footprints between 1972 and 2022 were reduced by 20% to measure the effect of their assumed change over time.

The change made for each parameter evaluated either increased or decreased the difference in input occurring over the 50 years to determine potential effects on the predicted differences in output metrics across the 50 years. A sensitivity index was determined as the absolute value of the percentage change in output over the percentage change in input times 100. An index near zero indicated very low sensitivity and a value near or greater than 100 indicated very sensitive to the input change. For interpretation, values less than 10 implied insensitive, 10–30 were moderately sensitive, 30–60 were sensitive and above 60 were highly sensitive.

## Results

### Historical trends

#### Feed production

Yields of the major feed crops used for cattle production increased over the past 50 years (Table 2; Table S1). Averaged over the US, corn grain yields increased by over 100%. Land area used for corn production also increased by 40%. This was primarily due to the use of corn grain in ethanol fuel production where only a portion was allocated to cattle production through the feeding of distiller’s grain. Corn silage yields increased by 78% with a 21% decrease in land area used for silage production. The portion of total corn land irrigated increased, likely due to increased production in the drier regions of the western US. Sorghum grain yields did not change much over this period, and the land area used to produce this grain decreased by 71% along with a decrease in the portion irrigated. Sorghum silage showed a trend for increased yield with over a 50% decrease in land area and a major increase in the portion irrigated. Wheat yields increased by over 50% with a decrease in land area and no change in the portion irrigated.

**Table 2:** Feed crop production and related resource data for the US in 1972 and 2022.

Crop characteristic	**Source** ^1^	Units	1972	2022	**Change,** ^2^ **%**	**R squared** ^3^
**Corn grain yield**	N	t DM/ha	4.5	9.4	110	0.89
**Corn grain area**	N	kha	24,770	34,604	40	0.64
**Irrigated corn grain area**	N	%	9.6	13.6	42	0.89^4^
**Corn silage yield**	N	t DM/ha	8.9	15.8	78	0.83
**Corn silage area**	N	kha	3,059	2,405	−21	0.31
**Irrigated corn silage area**	N	%	10.5	27.1	158	0.84
**Sorghum grain yield**	N	t DM/ha	3.0	3.6	21	0.15
**Sorghum grain area**	N	kha	5,470	1,574	−71	0.96
**Irrigated sorghum grain area**	N	%	18.2	7.1	−61	0.81
**Sorghum silage yield**	N	t DM/ha	8.1	10.4	29	0.39
**Sorghum silage area**	N	kha	361	154	−57	0.86^4^
**Irrigated sorghum silage area**	N	%	13.9	32.0	130	0.81
**Wheat yield**	N	t DM/ha	1.8	2.8	56	0.83
**Wheat area**	N	kha	26,506	16,341	−38	0.65
**Irrigated wheat area**	N	%	6.1	6.5	7	0.038
**Alfalfa hay yield**	N	t DM/ha	6.8	7.6	12	0.30
**Alfalfa hay area**	N	kha	10,800	6,747	−38	0.90
**Irrigated alfalfa hay area**	N	%	20.0	36.1	81	0.94
**Grass hay yield**	N	t DM/ha	3.6	4.6	28	0.63
**Grass hay area**	N	kha	10,742	15,141	41	0.73
**Irrigated grass hay area**	N	%	6.7	8.6	28	0.63
**Pasture and rangeland grazed**	N	kha	211,920	162,709	−23	0.96
**Irrigated pasture & range area**	N	%	0.70	0.67	−4	0.011
**Irrigation water use in the US**	N	cm	64	48	−26	0.88
**Corn land receiving N fertilizer**	E	%	96	98	3	0.29
**N fertilizer applied to corn**	E	kg/ha	135	167	24	0.51
**Corn land receiving P fertilizer**	E	%	89	76	−14	0.78
**P fertilizer applied to corn**	E	kg/ha	72	72	−1	0.19
**Wheat land receiving N fertilizer**	E	%	63	89	41	0.93^4^
**N fertilizer applied to wheat**	E	kg/ha	53	79	49	0.83^4^
**Wheat land receiving P fertilizer**	E	%	43	74	71	0.84
**P fertilizer applied to wheat**	E	kg/ha	41	37	−10	0.12
**Herbicide use on corn**	F	kg/ha	1.6	2.8	68	0.000
**Glyphosate use on corn**	F	kg/ha	0.0	0.9	—	0.88
**Insecticide use on corn**	F	g/ha	303	21	−93	0.69

1 Sources of data are [N] USDA NASS (2025), [E] USDA-ERS (2025), and [F] Fernandez-Cornejo (2014).

2 Positive numbers indicate an increase and negative values are a decrease over the 50-yr period.

3 Square of the correlation for the regression equation describing the relationship of annual values over time. This was a linear relationship unless designated as polynomial.

4 A polynomial relationship provided the best fit.

Forage crop yields did not show as much change as that of grain crops (Table 2; Table S1). Alfalfa hay yields showed a trend toward a small increase, with a major decrease in production area and a substantial increase in the use of irrigation. This reflected a shift from dry land production in the east to irrigated production in dryer regions of the west. Data for grass hay production indicated a stronger trend for a 28% increase in harvested yield with a 41% increase in land area and a 28% increase in irrigation use.

Beef cattle are the primary user of grazed pasture and rangeland with some use by horses, dairy cattle and other ruminant species. Land area used for grazing pasture and rangeland decreased by 23% with little change in the portion irrigated (Table 2). Pasture and rangeland yields over the 50-yr period were not well documented. Pasture yield increases have occurred on some operations through improved species and greater use of rotational grazing management, but the national impact of this improvement may be small (producer opinion). Rangeland yields have likely not increased and may have decreased due to decreasing precipitation and increasing temperatures in the western US (USDA-ARS 2025). For our LCA of beef production systems, little change in pasture and rangeland yields was assumed, but the sensitivity to this assumption was tested.

Throughout the 50-yr period, nearly all corn land received N fertilizer and most of that land received P fertilizer (Table 2). Application rates indicated a weak trend towards a small increase in N applied and no change in P application over time. For our LCA, N application rates for operations in the 1970s were reduced by 20% with no change in P application relative to 2022. Considering the large increase in yield, production in 2022 used 40–50% less fertilizer per unit of grain produced. Wheat land receiving fertilizer was less in the 1970s with a lower N application rate and little difference in P application rate (Table 2). For our LCA, much of the P fertilizer used on cattle operations in 2022 was assumed to come from the manure produced on those farms, ranches and feedlots. For lack of better information, we assumed the same for the 1970s.

Pesticide use on corn changed considerably over this period (Table 2). Herbicide use per unit of land increased to a peak in 1990 with a decrease toward 2000 and then little change to 2022 (USDA NASS 2025). Considering the increase in corn yield, this was a small decrease per unit of grain or forage produced. The major change was the adoption of glyphosate for weed control, which offset other herbicide use (Table 2; USDA NASS 2025). Insecticide use on corn peaked around 1980 and decreased considerably by 2010 (USDA NASS 2025). Genetic improvement through the development of insect resistance reduced insecticide use to minimal levels in current corn production systems.

#### Cattle production

The number of beef cows maintained as breeding stock decreased by 27% over the 50-yr period (Table 3; Table S2). The annual number harvested was highly variable however with little overall change (Table 3). The high variability, due to varying cattle prices and the need to destock created by weather, masked any long-term change in annual harvest rate. Also, an increase in replacement rate over the period partially offset the increase in cow numbers to provide a more consistent harvest rate. The annual number of dairy cows harvested was highly variable but trended down with the portion harvested increasing 38% (Table 3). This increase from 24% to 33% of the dairy herd harvested annually reflected an increase in dairy cow replacement rate. The dress weight, and therefore BW, of all cows harvested increased by 37% over the 50-yr period.

**Table 3:** Cattle characteristics and production data for the US in 1972 and 2022.

Animal characteristic	**Source** ^1^	Units	1972	2022	**Change,** ^2^ **%**	**R squared** ^3^
**Beef cows maintained**	N	Number	40,266,000	29,421,000	−27	0.73
**Calf birth weight**	A	kg	30	34	13	0.85^4^
**Weaning weight**	A	kg	205	282	38	0.98^4^
**Yearling weight**	A	kg	336	454	35	0.98^4^
**Beef cows harvested**	N	Number	3,725,000	2,984,000	−20	0.13
**Annual portion harvested**	N, C	%	9	10	12	0.039
**Dairy cows harvested**	N	Number	3,257,000	2,742,000	−16	0.10
**Annual portion harvested**	N, C	%	24	33	38	0.03
**All cows dress weight**	N	kg	215	295	37	0.94
**Beef cow replacement rate**	N, C	%	12	13	12	0.20
**Steers harvested**	N	Number	17,138,000	16,278,000	−5	0.16
**Steer dress weight**	N	kg	301	410	36	0.98
**Heifers harvested**	N	Number	9,860,000	9,807,000	−1	0.005
**Heifer dress weight**	N	kg	256	383	50	0.98
**Steers to heifers fed**	N, C	Ratio	1.76	1.67	−5	0.015
**Cattle harvested**	N, C	Number	34,727,000	32,323,000	−7	0.13
**Overall dressing**	N	%	59.3	60.9	2.7	0.49
**Beef breed harvest weight**	N, C	Gg	8,927	9,398	4.3	0.07
**Dairy breed harvest weight**	N, C	Gg	979	2,898	196	0.83
**Total harvest weight**	N	Gg	9,976	12,298	23	0.67
**Beef portion from dairy**	N, C	%	9.8	23.6	138	0.78
**Finishing phase in weight**	B, N, C	kg	286	342	20	0.42
**Finishing phase out weight**	B, N, C	kg	435	625	44	0.95
**Finishing days on feed**	B, C	days	127	197	55	0.76
**Finishing feed dry matter intake**	B, C	kg/day	16.6	21.5	30	0.82
**Finishing average daily gain**	B, C	kg/day	1.23	1.47	20	0.59
**Finishing gain to feed**	B, C	ratio	0.16	0.15	7	0.21

1 Sources of data are [N] USDA NASS (2025), [A] AAA (2025), [B] Betts and Harborth (2024), and [C] calculated from these sources.

2 Positive numbers indicate an increase and negative values are a decrease over the 50-yr period.

3 Square of the correlation for the regression equation describing the relationship of annual values over time. This was a linear relationship unless designated as polynomial.

4 A polynomial relationship provided the best fit.

Beef calf birth weights increased about 13% while weaning, yearling and steer dress weights increased about 35% (Table 3). The increase in dress weight of heifers was greater at 50% with the annual increase being very consistent and linear over the period (*R*^2^ = 0.98). The annual number of beef-breed steers and heifers harvested was relatively constant over the 50 years and the steer to heifer harvest ratio also did not change. Overall, the annual number of cattle harvested showed a trend for a small decrease. The overall dressing percentage also indicated a trend for a small increase.

The total annual CW produced increased by 23% (Table 3). The major source of this increase was culled animals from dairy farms, particularly calves raised and harvested for beef. With fewer but larger animals, the annual CW harvested from traditional beef breeds was highly variable and showed little change over the 50-yr period. Cattle from the dairy industry increased by 2-fold. In the early 1970s, dairy beef came from culled dairy cows as calves were not normally used for beef. As discussed above, dairy cull cow numbers did not change much over the period but there was a large increase in the production and finishing of cattle from dairy calves including those cross bred for beef production. The portion of beef coming from the dairy industry increased from about 10% in 1972 to 24% in 2022.

The size of feedlot finishing operations grew considerably over the 50-yr period with few having an inventory of more than 1,000 cattle in 1972 to 74% of the cattle finished on operations with an inventory greater than 2,500 cattle by 2022 (USDA NASS 2025). Finish weight grew steadily over the years with 2022 weight about 44% greater than that in the early 1970s (Table 3). According to the data of Betts and Harborth (2024), weights entering finishing operations have been variable, indicating a trend for about a 20% increase. Much of this variability was due to differences in the amount of time cattle spent in stocker or backgrounding operations before entering the finishing operation. Since yearling weight increased about 35% over the 50 years, the lower increase in entering weight indicated that more cattle entered the finishing operation at a lower weight and younger age in 2022. As a result, the days-on-feed increased by 55% while time on pasture or rangeland decreased. Average daily gain during the finishing phase increased about 20%, while feed dry matter intake increased by about 30%. Overall, the gain-to-feed ratio did not change.

### Model results

The first step of the modeling procedure was to verify the simulation results of beef production in 2022. Feed consumption to produce traditional beef breeds (21.8 kg DM/kg CW) was 2% less than that previously reported for 2017 (Rotz et al. 2019) verifying good agreement. Intensities of CO_2_e emissions and fossil energy use were 9% less than the previous values reported for traditional beef breeds and blue water use and reactive N losses were 6% and 10% greater. When cattle from dairy operations were included, CO_2_e emissions and energy use were 8% and 6% less than those of the previous study. Blue water use was 2% greater and reactive N losses were 8% greater. The minor improvements reflect continuing gains in production efficiency and greater use of cull animals from dairy production, but this was confounded by minor changes due to continuing development of the model. The most notable model change was in representing NH_3_ volatilization following urea fertilizer application and the slow transformation and release of NH_3_ from crop and pasture lands throughout the year (Rotz et al. 2021). Another change involved further development and verification of irrigation practices (Castano-Sanchez et al. 2025). All data for 2022 production were within the uncertainty reported in the previous study for production in 2017 (Rotz et al. 2019).

#### Feed consumption

With the decrease in animal numbers offset by the increase in animal size, total feed consumption by traditional beef cattle decreased 8% over the 50-yr period (Table 4). This included a 16% reduction in grazed forage consumption along with 15% increases in the use of grain and other concentrate feeds. When the large increase in the use of cull dairy animals and beef breeds bred to dairy cows was included, little change was found in total feed consumption by all cattle used for beef (Table 4). This was due to greater use of harvested forage and the feeding of more grain and other concentrates to grow and finish calves from dairy operations compared to traditional beef cattle production.

**Table 4:** Changes in feed, energy, and water use and various environmental impacts of beef cattle production from 1972 to 2022.

		Traditional beef cattle			Traditional beef plus dairy cull cattle		
	Units	1972	2022	**Change,** ^1^ **%**	1972	2022	**Change,** ^1^ **%**
**Total feed intake**	Gg DM	230,649	212,065	−8	243,185	239,741	−1
**Grazed forage**	Gg DM	158,356	133,584	−16	159,260	133,584	−16
**Harvested forage**	Gg DM	32,369	32,843	1	40,145	46,322	15
**Grain**	Gg DM	26,591	30,529	15	29,060	38,933	34
**Other concentrates**	Gg DM	11,978	13,776	15	13,508	19,467	44
**Fuel**	ML	3,279	2,386	−27	3,481	2,719	−22
**Natural gas**	Gg	305	423	39	347	518	49
**Electricity**	GW	10,415	4,537	−56	11,575	6,151	−47
**Ammonia emission**	Gg	1,107	1,273	15	1,231	1,640	33
**Nitrous oxide emission**	Gg	195	157	−20	208	191	−8
**Methane emission**	Gg	5,175	4,502	−13	5,508	5,089	−8
**Non-methane VOC emission**	Gg	71.7	78.6	10	88.3	107	21
**Nitrogen runoff**	Gg	23.0	8.7	−62	25.6	14.1	−45
**Nitrogen leached**	Gg	429	203	−53	487	272	−44
**Phosphorus loss**	Gg	18.3	6.8	−63	20.4	9.0	−56
**Erosion of sediment**	Tg	30.5	15.8	−48	35.2	19.8	−44
**Life cycle impacts**							
**Fossil energy use**	PJ	727	456	−37	793	588	−26
**CO_2_e emission**	Tg	263	206	−21	281	245	−13
**Blue water use**	Tg	32,696	20,756	−37	34,532	25,140	−27
**Reactive nitrogen loss**	Gg	1,934	1,652	−15	2,122	2,105	−1
**Land use**	Mha	218	194	−11	220	196	−11
**Grazing land use**	Mha	207	185	−11	207	185	−11
**Cropland use**	Mha	11.1	8.7	−23	13.0	11.1	−14

1 Positive numbers indicate an increase and negative values are a decrease over the 50-yr period.

Considering the substantial increase in CW produced over the period, greater changes were found per unit of beef produced (Table 5). Total feed consumption per unit of CW by traditional beef breeds decreased 17% while that including animals from dairy decreased 22%. Due to the large increase in the use of dairy cull animals, the increased CW from dairy created a major decrease in the intensity of feed consumption, i.e., this reduced the feed needed to maintain beef cows and bulls used to produce beef calves. The finishing of dairy animals used a longer finish period with the feeding of less forage and more concentrate, which also reduced total feed consumption per unit of CW produced.

**Table 5:** Changes in the intensity of feed, energy, and water use and various environmental impacts of beef cattle production from 1972 to 2022.

		Traditional beef cattle			Traditional beef plus dairy cull cattle		
	Units	1972	2022	**Change,** ^1^ **%**	1972	2022	**Change,** ^1^ **%**
**Total feed intake**	kg DM/kg CW	26.2	21.8	−17	24.4	19.0	−22
**Grazed forage**	kg DM/kg CW	18.0	13.7	−24	16.0	10.6	−34
**Harvested forage**	kg DM/kg CW	3.7	3.4	−8	4.0	3.7	−9
**Grain**	kg DM/kg CW	3.0	3.1	4	2.9	3.1	6
**Other concentrates**	kg DM/kg CW	1.4	1.4	4	1.4	1.5	14
**Fuel**	ml/kg CW	372	245	−34	349	216	−38
**Natural gas**	g/kg CW	34.6	43.4	26	34.8	41.1	18
**Electricity**	W/kg CW	1,181	466	−61	1,160	489	−58
**Ammonia emission**	g/kg CW	123	131	4	121	130	6
**Nitrous oxide emission**	g/kg CW	22.1	16.1	−27	20.8	15.1	−27
**Methane emission**	g/kg CW	587	463	−21	552	404	−27
**Non methane VOC emission**	g/kg CW	8.1	8.1	−1	8.9	8.5	−4
**Nitrogen runoff**	g/kg CW	2.6	1.1	−57	2.6	1.1	−56
**Nitrogen leached**	g/kg CW	49	21	−57	49	22	−56
**Phosphorus loss**	g/kg CW	2.1	0.7	−66	2.0	0.7	−65
**Erosion of sediment**	g/kg CW	3.5	1.6	−53	3.5	1.6	−55
**Life cycle impacts**							
**Fossil energy use**	MJ/kg CW	82.4	46.8	−43	79.5	46.7	−41
**CO_2_e emission**	kg/kg CW	29.8	21.2	−29	28.2	19.5	−31
**Blue water use**	kg/kg CW	3,708	2,133	−42	3,462	1,997	−42
**Reactive nitrogen loss**	g/kg CW	219	170	−23	213	167	−21
**Land use**	m^2^/kg CW	248	199	−20	221	156	−29
**Grazing land use**	m^2^/kg CW	235	190	−19	208	147	−29
**Cropland use**	m^2^/kg CW	12.6	8.9	−29	13.0	8.8	−32

1 Positive numbers indicate an increase and negative values are a decrease over the 50-yr period.

#### Energy use

Total life cycle fossil energy use in the production of traditional beef cattle was found to decrease 37% and by 26% when cattle from dairy farms were included (Table 4). This was primarily due to reductions in fuel and electricity use through more efficient equipment. These reductions were partially offset by greater use of natural gas (which could include propane) for irrigation and steam flaking of grain. When expressed per unit of CW produced, total life cycle fossil energy use decreased 41% over the 50-yr period with large decreases in fuel and electricity use and an increase in gas use (Table 5).

#### Water use

The change in blue water use in cattle production was more challenging to predict than the other metrics considered. Irrigation of feed crops used most of the water, so irrigation efficiency had a strong influence on water use. For irrigated feed crops produced on farms and feedlots, we assumed about 75% of the irrigation in 1972 was done by furrow or flood irrigation (USDA NASS 2025) with more efficient sprinkler irrigation dominant in 2022. This increased water use efficiency in 2022 but also increased energy use. Following these assumptions, total life cycle blue water use in producing traditional beef breeds decreased 37% over the period. With cattle from dairy systems included, the reduction was lower at 27% (Table 4). The reduction over all cattle was 42% when expressed per unit of CW produced (Table 5).

#### Land use

Total and grazing land used in beef cattle production were found to decrease about 11% (Table 4). This was primarily due to the grazing of fewer animal units in 2022 compared to 1972. Cropland was found to have a larger percentage decrease due to the large increase in corn yields along with smaller yield increases of other crops (Table 2). With greater yields, less land was used to produce the feed required. Cropland decreased by 23% for producing traditional beef breeds and 14% over all cattle used for beef production (Table 4). Reductions in land use were greater when expressed per unit of beef produced (Table 5). For traditional beef breeds, total land use per unit of CW decreased by 20% and cropland decreased by 29%. With cattle from dairy operations included, reductions were 29% and 32%, respectively.

#### Carbon emissions

Total life cycle CO_2_e emissions associated with the production of traditional beef breeds decreased 21% and by 13% when cattle from dairy systems were included (Table 4). Over all cattle used for beef, this included 8% reductions in CH_4_ and N_2_O emissions. These reductions resulted from fewer cattle used to produce beef, but this was partially offset by feeding and maintaining larger animals. Expressed per unit of CW produced, CO_2_e emissions for all beef cattle decreased by 31% with 27% decreases in CH_4_ and N_2_O emissions (Table 5).

#### Reactive nitrogen losses

Considering all forms of reactive N lost in the life cycle of beef cattle production, little change was found in total loss over the 50-yr period (Table 4). Although a 15% reduction was found in producing traditional beef breeds, there was no change when cattle from dairy production were included. Compared to the time spent on pasture in cow calf systems, cattle from dairy farms spent their full life cycle on feedlots or in barns creating greater NH_3_ emissions. Beef breeds also spent less time on pasture and more time on feedlots in 2022. This increase in NH_3_ emissions was offset by predicted reductions in N runoff and leaching (Table 4). Runoff and leaching losses decreased through the shift toward greater use of more efficient sprinkler irrigation and the reduction in use of intensive tillage systems. When expressed per unit of beef produced, reactive N loss decreased by 21% (Table 5). Ammonia emissions still showed a small increase while runoff and leaching losses showed greater decreases (Table 5).

#### Other emissions

On farms and feedlots, VOC primarily comes from silage and long-term manure storage (Bonifacio et al. 2017; Rotz et al. 2022). With the increase in size of cattle finishing operations, use of silage stored in bunkers or piles has increased. The exposed surface of these piles and feed bunks provide sources for non-methane VOC emissions (Table 4). Larger manure storage piles and runoff catchments also provide sources for these emissions. An increasing portion of that allocated to cattle coming from dairy farms has also contributed. Over the 50-yr period, our assessment indicates a 21% increase in annual VOC emissions related to beef cattle production. When expressed per unit of CW produced though, there has been little change (Table 5).

Sediment erosion and P runoff losses can be of concern, particularly in eastern regions of the US, for their contribution to the pollution of surface waters as well as the loss of a valuable soil resource (Kleinman et al. 2019). Our model procedure estimated that erosion related to feed production for beef cattle decreased 44% over this period (Table 4) or 55% when expressed per unit of CW produced (Table 5). Adoption of less intensive tillage systems and the use of less cropland to produce feed has led to this reduction. The predicted reduction in P loss was greater, at around 60% (Tables 4 and 5). As with erosion, less land needed to produce feed and reduced tillage operations contributed to this reduction along with less use of flood and furrow irrigation and increased use of cover crops.

### Sensitivity analysis

Parameter changes made in the sensitivity analysis related to crop and pasture production had relatively small effects on the change in life cycle metrics over the 50-yr period (Table 6). The change in stocking rate had greater effect than most of the parameter changes. Land use was moderately sensitive to stocking rate where the 10% reduction in the difference between 1972 and 2022 reduced the difference in land use by 23%, i.e. the 11% reduction of the original assessment was reduced to 8.5%. Other metrics showed relatively low sensitivity with indexes less than 10. Reducing the difference in pasture yield or the legume content of the pasture sward did not have much effect on change in life cycle metrics (Table 6). Feed consumption and reactive N losses were moderately sensitive to the greater difference in pasture quality due to a decrease in forage intake and increase in required concentrate feeding over the period. Reducing the difference in pasture irrigation primarily affected water use but all metrics were relatively insensitive to this change.

**Table 6:** Sensitivity of changes in life cycle metrics between 1972 and 2022 to changes in parameters used to model beef production systems in the US. Sensitivity index^1^ values less than 10 imply insensitive, 10–30 are moderately sensitive, 30–60 are sensitive and above 60 are highly sensitive.

Parameter change	Feed consumption	Fossil energy use	CO_2_e emission	Reactive nitrogen loss	Water use	Land use
**Grazing area/stocking rate** ^2^	0.8	4.1	3.0	9.3	0.5	23
**Pasture yield** ^3^	0.2	1.4	1.7	3.8	2.0	0.4
**Pasture quality** ^4^	11	1.9	3.7	42	0.8	0.5
**Pasture legume content** ^5^	0.1	0.4	0.0	1.0	0.5	0.1
**Pasture irrigation** ^6^	0.1	0.3	0.1	0.8	8.4	0.1
**Crop & pasture N fertilizer** ^7^	0.2	3.7	5.0	10	1.5	0.3
**Stocker/background period** ^8^	11	5.4	0.5	0.8	4.0	1.1
**Finish period** ^9^	10	10	11	32	7.2	1.5
**Calving rate** ^10^	7.7	1.5	3.8	4.8	2.0	0.4
**Replacement rate** ^11^	1.6	0.5	1.0	1.2	0.7	0.1
**Purchased feed footprints** ^12^	0.0	5.1	1.4	5.0	4.2	0.0
**Protein feeding** ^13^	1.9	14	4.6	11	4.9	2.6
**Forage content in finishing diet** ^14^	0.4	0.2	0.1	0.4	0.4	0.1

1 The sensitivity index is the absolute value of the percentage change in the difference between 1972 and 2022 over the percentage change in the assumed parameter.

2 Difference in stocking rate between 1972 and 2022 reduced by 10%.

3 Difference in pasture yield between 1972 and 2022 reduced by 10%.

4 Pasture quality in 1972 reduced (10% less CP and 10% greater NDF).

5 Legume content of pasture sward in 1972 reduced by 20%.

6 Pasture irrigation rate in 1972 reduced by 15%.

7 Nitrogen fertilizer use in 1972 increased 10%.

8 Stocker period in 1972 increased by 1 month.

9 Finish period in 1972 increased by 1 month.

10 Calves weaned in 1972 decreased from 84.4% to 82.8 with 2% more cows.

11 Difference in cow replacement rate between 1972 and 2022 reduced by 50%.

12 Difference in purchased feed footprints between 1972 and 2022 reduced by 20%.

13 Protein content of finishing diet in 1972 increased by 10%.

14 Forage content of finishing diet in 1972 increased by 10%.

Parameter changes related to animal management showed greater sensitivity, but effects were still small in most cases (Table 6). Increasing the difference in the stocker period by 1 month increased the effect on feed consumption by 11%, i.e., 0.9% more feed was used in 2022 relative to 1972 compared to the base simulation. The change in stocker period showed relatively small effects for fossil energy and water use. Increasing the difference in finishing period increased the change in all life cycle metrics over the 50 years (reduced the original benefit) with moderate sensitivity on feed consumption, fossil energy use, CO_2_e emission, and water use and greater sensitivity on reactive N losses. Changes in all metrics were relatively insensitive to an increased difference in calving rate, but the increase created a small increase in required feed consumption, CO_2_e emission and reactive N losses (Table 6). Reducing the difference in annual replacement rate of cows over the period did not have much effect on the life cycle metrics of the base simulation.

Fossil energy use and reactive N loss were moderately sensitive to greater protein in diets in 1972 (Table 6). Feeding more protein used more high protein concentrates which required more energy to produce. With more protein fed, more N was excreted leading to greater N losses, particularly greater NH_3_ emission from feedlot manure (Bonifacio et al. 2015). These increased impacts in 1972 created greater beneficial changes over the 50-year period. Reducing the difference in footprints of all purchased feeds between 1972 and 2022 had a relatively small impact, i.e., LCA metrics were relatively insensitive to this change.

The sensitivity analysis illustrates that our comparison of beef cattle production in 2022 to 1972 was not highly sensitive to any of the input assumptions considered. Those considered were factors with less certainty in the original definitions of the production systems. Although the comparisons across time may be more sensitive to over input parameters, those parameters were also better documented with available data.

## Discussion

Several studies have used LCA procedures to assess beef production systems in the US. A few have also been made in Canada where similar production practices are used. Due to differences among models and assumptions used, quantifiable comparisons of results cannot be made. Differences in system boundaries, functional units, model relationships, inventory values, etc. confound the comparisons among published studies. Comparisons are still useful though to ensure similar results and trends across studies.

Most previous studies have emphasized CO_2_e emissions, but other metrics have sometimes been included. In early work, Phetteplace et al. (2001) determined a CO_2_e emission intensity of 15.5 kg/kg BW (25 kg/kg CW) for a typical US beef cattle production system. Stackhouse-Lawson et al. (2012) used IFSM to determine the intensity of a British breed production system in California to be 22.6 kg CO_2_e/kg CW. In another study using IFSM to assess an intensively managed cattle production system at a research facility, Rotz et al. (2013) found an average annual value of 10.9 ± 0.6 kg CO_2_e/kg BW (17.6 ± 1.0 kg CO_2_e/kg CW). Other studies have documented emission intensities for typical US production systems of about 24 and 26 kg CO_2_e/kg CW (Pelletier et al. 2010), 17 kg CO_2_e/kg CW (Roop et al. 2014), and 23 kg CO_2_e/kg CW (Lupo et al. 2013). These previous studies help support our modeling of US beef cattle production in 2022, and that the predicted emission intensity of 22.1 kg CO_2_e/kg CW is representative of national practices.

Rotz et al. (2013) also quantified a few additional metrics of sustainability for a British breed cattle production system using IFSM. The intensity of fossil energy use was determined to be 26.5 ± 4.5 MJ/kg BW (42.7 ± 7.3 MJ/kg CW) which was within the range of that found in the current assessment for 2022. Water required was much greater than that of the current study at 2,790 ± 910 L/kg BW (4,500 ± 1,470 L/kg CW) due to heavy use of irrigated crop and pasture production in this specific production system (Rotz et al. 2013).

A few LCA studies of cattle production in Canada also help support our assessment of US cattle production. In an assessment of a production system in Western Canada, Beauchemin et al. (2010) estimated the CO_2_e emission intensity of beef production at 22 kg/kg CW, essentially the same as that we determined for 2022 in our current LCA. In a more comprehensive LCA of beef production in Canada, Aboagye et al. (2024) considered 7 metrics of environmental sustainability. The national CO_2_e emission intensity was about 17 kg/kg CW for beef breed cattle production and 16 kg/kg CW including calves and cattle from dairy farms, both of which were about 20% less than the US values we determined for 2022 (Table 5). Blue water use (1,084 L/kg CW) and fossil fuel use (converted to 28 MJ/kg CW) were about half that determined in our current LCA for 2022.

Although LCA studies have produced a range in results, these comparisons generally support our results for recent beef cattle production in the US. Reasons for differences between studies are difficult to determine and are often due to many factors including the use of different models, modeling procedures and assumptions. Differences in climate and production practices contribute to the differences in metrics across studies, but these effects are not known without using the same models and procedures across systems. Models and standard procedures continue to develop, causing differences among studies over time. Standardized procedures also allow local assumptions which can affect and even bias the results obtained. Considering these issues, changes over time cannot be made based upon different studies, i.e., the same model, procedures and overall assumptions must be used.

Relatively few studies have used LCA to determine changes in US beef cattle production over time. Although not an LCA, the most comprehensive assessment was that of Capper (2011). Changes in environmental impacts were compared over a 30-yr period from 1977 to 2007. On an intensity basis, beef was reported to be produced using 30% fewer animals, 19% less feed, 12% less water, and 33% less land. This led to a 16% reduction in the CO_2_e emission of beef cattle production. Our LCA was similar but more conservative on some metrics with 21% and 29% decreases in feed consumption and land use over 50 years. Our LCA also determined greater reductions for water use (41%) and CO_2_e emissions (29%) over the longer period.

Rotz et al. (2013) used the IFSM and LCA procedures to assess changes in a specific US beef cattle production system from 1970 to 2011. Over the 40-yr period, the intensity of CO_2_e emissions of the cattle produced decreased 6% with no change in the fossil energy use, a 3% reduction in reactive N losses, and a 6% reduction in the real cost of production. Due to increased use of irrigated corn production for this specific operation, the blue water footprint increased 42%. These reported changes over time were quite different from those of the present study due the much different management changes made over the 40 years on the research facility modeled in the previous study (Rotz et al. 2013).

Klopatek and Oltjen (2022) quantified the change in water use in US beef cattle production over a 28-yr period from 1991 to 2019. They found a 29% reduction in total water use which was similar to that we determined over the 50-yr period. They reported a 38% reduction in the intensity of water use which also compared closely to the 41% reduction determined in our LCA. It may be that most of the change in water use over the past 50 years has occurred during the past 28 years, strengthening the comparison of the two independent studies.

A few studies have also investigated changes in the environmental impacts of beef cattle production in Canada. Vergé et al. (2008) found a 37% reduction in the CO_2_e emissions over the 25-yr period from 1981 to 2006. This assessment was expanded by Desjardins et al. (2012) to include soil C change and dairy allocation which produced a 48% reduction. Legesse et al. (2016) studied the period from 1981 to 2011 and found that Canadian beef production required 29% fewer animals in the breeding herd and 24% less land with a 14% decline in the intensity of CO_2_e emissions. Legesse et al. (2018) assessed the change in water use over the same period and found a 20% decline in the intensity of use.

Looking to the future, LCA can be used to evaluate production changes to improve the sustainability of beef production. Pelton et al. (2024) used LCA to evaluate 42 practices spanning the supply chain from crop and livestock production to processing. They found that the potential to reduce CO_2_e emission across the beef sector ranged up to 30% under ubiquitous adoption assumptions. Most promising opportunities were in the grazing stage. Their assessment indicated promising locations and practices for mitigation and an upper-end theoretical potential for mitigation in the beef industry.

When evaluating CO_2_e emissions from beef systems, effects of soil C storage are of interest. With transitions in management such as reduced tillage, use of cover crops and improved grazing management, soils can sequester C for years until a new equilibrium is established. Quantifying these effects has been challenging. Conversion of cropland to pasture and intensifying grazing practices on pastureland has been found to sequester 0.5 to 3 Mg C/ha per year for up to 15 years or more (Franzluebbers and Stuedemann 2009; Teague et al. 2016) while sequestering additional C in drier rangeland soils appears difficult (Henry et al. 2024). In annual cropping systems, reduced tillage practices and use of cover crops can increase soil C storage by 0.2 to 0.5 Mg/ha per year (Franzluebbers 2005; Teague et al. 2016), but intensifying cropping systems may also reduce soil C (Gamble et al. 2021).

There is much uncertainty in considering soil C changes related to cattle production over the past 50 years, but a brief example can illustrate the potential effect. If reduced tillage practices and the use of cover crops in the eastern region sequestered 10 Mg/ha (0.5 Mg/ha per year over 20 years) on 70% of the cropland with a similar sequestration rate obtained through improved grazing management on 20% of the pastureland, a total of 108 Tg of C was stored. This would offset about 400 Tg of CO_2_e or 3% of the total CO_2_e emission related to beef cattle production in the US over the 50 years. These values are very speculative but provide an illustration of the potential long-term impact of C sequestration. Regardless of the potential of sequestering additional soil C, cattle grazing has contributed to retaining and maintaining soil C storage. Continuing to retain this soil C is an important benefit of maintaining cattle grazing systems.

As we pursue further improvement in the sustainability of beef production, LCA provides a valuable tool for quantifying improvements and directing the industry toward the most promising strategies. A comprehensive LCA is needed to summarize and integrate the many metrics associated with the sustainability of beef. Additional metrics could include fossil resource scarcity, freshwater ecotoxicity, freshwater eutrophication, human toxicity, ionizing radiation, marine ecotoxicity, and terrestrial ecotoxicity (Putman et al. 2023). Much emphasis has been placed on CO_2_e emissions in recent years, but sustainability is much broader and requires a more comprehensive assessment.

## Conclusions

Compared to 1972, 14% fewer but larger cattle were harvested in 2022 producing 23% more beef CW. Nearly the same total feed was consumed to produce beef in 2022 as used in 1972, but 9% less forage and 39% more grain and byproduct feeds were used to meet nutrient requirements. Increases in crop yields, particularly corn yields, led to 11% reductions in cropland and total land use over the 50-yr period. These improvements in feed and cattle production led to the following improvements in the life cycle of cattle production from 1972 to 2022:

1. Blue water use was reduced by 25% through more efficient irrigation along with greater crop yields and reduced land use, and per unit of CW produced, the reduction was 41%.
2. Fossil energy use was reduced by 23% through larger and more efficient equipment, increased crop yields and other efficiency improvements, which gave a 39% reduction per unit of CW.
3. Carbon dioxide equivalent emissions in beef cattle production decreased 11% including a 6% decrease in CH_4_ emissions and 5% decrease in N_2_O emissions. Per unit of CW, these reductions were 29%, 26% and 25%, respectively.
4. Reactive N losses from cattle production showed little change due to a 38% increase in NH_3_ emissions offset by decreases in N runoff, leaching and denitrification. Per unit of CW, reactive N losses were reduced 19% with a 10% increase in NH_3_ emissions.

## Supplementary Material

skag169_Supplementary_Data

## Data Availability

The Integrated Farm System Model (USDA-ARS 2026) used in the assessment is available at https://www.ars.usda.gov/northeast-area/up-pa/pswmru/docs/integrated-farm-system-model. Supplementary data supporting the modeling work is available at Journal of Animal Science online.

## Abbreviations

BW: body weight
CO_2_e: carbon dioxide equivalent
CW: carcass weight
DM: dry matter
IFSM: Integrated Farm System Model
LCA: life cycle assessment
N: nitrogen
VOC: volatile organic compound
